# Arteriolar Collapse and Haemodynamic Incoherence in Shock: Rethinking Critical Closing Pressure

**DOI:** 10.3390/jpm16020078

**Published:** 2026-02-01

**Authors:** Ashley Miller, Philippe Rola, Rory Spiegel, Korbin Haycock

**Affiliations:** 1 Shrewsbury and Telford Hospitals, Shrewsbury SY3 8XQ, UK; 2 Intensive Care Unit, Santa Cabrini Hospital, CIUSSS EMTL, University of Montreal, Montreal, QC H1T1P7, Canada; philipperola@gmail.com; 3 Departments of Critical Care and Emergency Medicine, Medstar Washington Hospital Center, Washington, DC 20010, USA; rspiegs@gmail.com; 4 Loma Linda University Medical Center, Loma Linda, CA 92354, USA; khaycockmd@hotmail.com; 5 Desert Regional Medical Center, Palm Springs, CA 92262, USA; 6 Departments of Emergency Medicine, Riverside University Health System Medical System, Moreno Valley, CA 92555, USA

**Keywords:** critical closing pressure, perfusion pressure, autoregulation, arteriolar collapse, haemodynamics, shock, incoherence, microcirculation

## Abstract

Critical closing pressure (CCP) and the vascular waterfall have long been used to explain perfusion failure in shock, yet their physiological meaning has been inconsistently interpreted. CCP is frequently treated as a continuous downstream pressure and inserted into formulas such as mean arterial pressure (MAP) − CCP, implying that a collapse threshold behaves like an opposing pressure even when vessels remain open. Drawing on classical vascular mechanics, whole-bed flow studies, microvascular models, and contemporary clinical physiology, we show that this interpretation is incorrect. Tone-dependent arteriolar collapse does not behave as a Starling resistor: CCP is a threshold at which smooth-muscle tension exceeds intraluminal pressure and vessels close, not a pressure governing flow in patent vessels. Perfusion becomes heterogeneous because different vascular beds reach their collapse thresholds at different pressures (via excessive tone, extrinsic compression, or profound hypotension), disconnecting macro-haemodynamics from microcirculatory flow. This explains why systemic variables such as MAP and systemic vascular resistance (SVR) may appear adequate even while tissues are under-perfused, a phenomenon now termed haemodynamic incoherence. Reframing CCP as a binary collapse threshold resolves longstanding contradictions in the literature, clarifies why MAP-centred targets often fail, and unifies the behaviour of shock states within a four-interface model of circulatory coupling. Therapeutically, the aim is not to “restore a waterfall” but to reopen closed vascular territories by lowering excessive tone, relieving external pressure, or raising truly low arterial inflow. This mechanistic reinterpretation provides a more coherent, physiologically grounded approach to personalised perfusion management in critical illness.

## 1. Introduction—Clearing the Confusion

Few ideas in circulatory physiology have caused as much persistent misunderstanding as critical closing pressure (CCP) and the so-called vascular waterfall. Both concepts appear frequently in the literature, but their definitions vary widely and are often contradictory.

Over time, this inconsistency has spawned a cascade of related fallacies. First, CCP has been portrayed as an arterial downstream pressure, directly analogous to central venous or mean systemic pressure (Pms). This, in turn, produced the modern definition of tissue perfusion pressure (TPP) as mean arterial pressure (MAP) − CCP, implying that a conditional collapse threshold behaves as a standing back-pressure even in open-flow states [1,2]. More recently, the vascular waterfall metaphor—originally developed for passive collapsible tubes—has been exported into arteriolar physiology. Some authors have attempted to “manipulate” this waterfall—for example, by pharmacologically widening the gap between CCP and Pms—in the hope of improving capillary flow [3,4]. In each case, the same conceptual error is repeated: confusing the conditions that create collapse with the normal physiology we aim to restore and treating a threshold phenomenon as if it were a continuous downstream pressure.

Such contradictions have inevitably led to scepticism about whether CCP and the vascular waterfall have any real clinical relevance. In truth, CCP is both real and important—but only under specific conditions. When correctly understood, it explains why tissue hypoperfusion can persist despite apparently adequate global haemodynamics—the phenomenon now termed haemodynamic incoherence [5,6]. We interpret this as loss of flow continuity across Interface 2 (the macro-to-micro transition), where macro-variables no longer predict microcirculatory flow. This mechanism is distinct from endothelial dysfunction, glycocalyx injury, and microthrombotic flow impairment described in sepsis, but in clinical practice these processes frequently coexist. The present framework isolates a pressure- and tone-dependent cause of incoherence without implying that other microcirculatory mechanisms are absent. Seen in this light, CCP reframes perfusion failure as a patient- and organ-specific physiological state rather than a universal haemodynamic target, providing a mechanistic basis for personalised perfusion management.

The aim of this article is not to deny CCP or collapse phenomena but to place them in their proper physiological context. We revisit the basic pressure–flow relationships, define CCP precisely as a tone-dependent arteriolar threshold, and separate this from the passive Starling resistor “waterfall” that applies to veins and zone-1 lung but not to arteriolar behaviour in shock. We then examine how these concepts illuminate organ-specific vulnerability, the limitations of systemic vascular resistance, and the management of shock states.

To frame these ideas, we use a four-interface model of the circulation, which views perfusion as a series of coupled pressure–flow relationships rather than a single driving gradient [7]. These interfaces map directly onto established physiological constructs: Interface 1 couples the left ventricle to the arterial system, where contractility and afterload determine the pressure head that drives flow. Interface 2 links the macro- to the microcirculation, where arteriolar tone and CCP govern whether vessels remain open or collapse. Interface 3 spans the capillary–venous junction, where flow is described by Guytonian venous return mechanics (Pms–RAP)/RVR where RAP denotes right atrial pressure and RVR resistance to venous return. Interface 4 connects the right ventricle to the pulmonary arteries and determines venous pressure and return; efficient coupling keeps RAP low, while raised intrathoracic pressure or RV failure elevates it and drives congestion upstream. Pathology or therapy can “uncouple” any of these interfaces by lowering arterial pressure, raising vascular tone, or increasing external pressure until local transmural pressure reaches the CCP and arterioles close—producing the haemodynamic incoherence so often seen in shock.

## 2. Perfusion Gradients in Normal Physiology

Blood flow in the intact circulation is maintained by three inter-related pressure gradients:

1. From arteries to arterioles:

Flow here is governed by metabolic demand. Arterioles adjust their tone through autoregulation, maintaining regional flow across a wide range of mean arterial pressures (MAP) [2,8,9].

2. Across the capillary bed—from arterioles to venules:

Capillary flow is driven by the pressure drop across the bed, determined by both arteriolar and venular pressures, and is modulated by upstream arteriolar tone until autoregulation fails.

3. From venules to the right atrium:

Venous return depends on the gradient between mean systemic pressure and right atrial pressure, divided by the resistance to venous return [10,11].

The term tissue perfusion pressure is often used in the literature to describe the driving pressure for microcirculatory flow and is frequently defined as TPP = MAP − CCP [1]. This formulation, however, treats CCP as if it were a continuous opposing pressure. In reality, CCP becomes relevant only once arterioles have collapsed; in open vascular beds, arteriolar pressure lies well above the collapse threshold, and flow is governed by autoregulation and by the arteriolar–venular pressure drop—not by subtracting CCP from MAP. Treating CCP as a fixed back-pressure, therefore, mistakes a conditional threshold for a standing variable.

A related misconception is the idea that capillary perfusion can be expressed as “CCP minus venous pressure,” as if CCP were the inflow pressure to the microcirculation. This is physiologically impossible. CCP is not an input pressure; it is a collapse threshold at the arteriolar segment. As long as the arterioles remain open, flow is governed by the arteriolar–venular pressure gradient and by arteriolar tone—not by subtracting venous pressure from CCP. Treating CCP as the driving pressure conflates a boundary condition with the pressure head that actually feeds the capillary bed, producing a quantity with no physiological meaning.

## 3. Autoregulation, Pressure Dependence, and the CCP Threshold

A frequent source of confusion is the assumption that the lower limit of autoregulation and the critical closing pressure describe the same event [1,12]. In reality, they are distinct physiological thresholds. Autoregulation is governed by the proximal arteriolar segment—the part of the microcirculation that contains smooth muscle and modulates tone [13]. Pressures here remain substantially higher than in distal arterioles or capillaries. As MAP falls, proximal arteriolar pressure eventually drops below the autoregulatory limit (typically around 60 mmHg), at which point arterioles become maximally dilated and flow becomes pressure-dependent, even though the vessels remain open. Only at much lower pressures (approximately 20–30 mmHg) does local arteriolar pressure approach the CCP, the point at which the arteriolar segment collapses and flow ceases entirely. This creates an intermediate pressure-dependent zone between the end of autoregulation and the onset of collapse: flow tracks MAP directly here, but CCP does not yet determine perfusion. Conceptually, vascular behaviour progresses from autoregulated flow, to pressure-dependent flow with open arterioles, to CCP-limited non-conductance once the collapse threshold is crossed.

As long as vessels remain open, perfusion is determined by the normal cascade of pressure gradients—arterial to arteriolar, arteriolar to venular, and venular to right-atrial—not by any putative “MAP − CCP” relationship. CCP becomes relevant only when local pressure falls far enough to collapse the arterioles [14,15,16].

## 4. Critical Closing Pressure—What It Is and What It Is Not

Critical closing pressure is the arterial pressure below which a vessel collapses and flow ceases. It is not a fixed standing pressure to be subtracted from MAP but a dynamic threshold that emerges when intraluminal pressure falls below the combined opposing forces of vascular smooth-muscle tone and external tissue pressure [17,18]. In practical terms, as long as local arterial pressure remains above CCP, the vessels stay open and perfusion continues; once pressure falls below CCP, the vascular segment collapses and flow stops. Bedside estimation is possible experimentally—for example, using inspiratory-hold manoeuvres after cardiac surgery [14]—but not feasible in routine clinical practice.

Baseline physiological CCP in healthy or low-tone states is generally around 20–30 mmHg when measured under true no-flow conditions [15]. In vasodilatory states such as sepsis, CCP falls and a lower pressure is required to maintain vascular patency. With intense vasoconstriction, raised intracranial or intrathoracic pressure, or severe tissue oedema, CCP rises, and a higher intraluminal pressure is required to keep arterioles open [19]. In critical illness, CCP often exceeds the physiological range: high sympathetic tone, exogenous catecholamines, raised intrathoracic pressure, and interstitial oedema all elevate the pressure required to prevent collapse [20].

These physiological shifts are only part of the story. Reported CCP values also differ because measurement techniques capture different flow states. True no-flow measurements during circulatory arrest typically yield CCP values around 20–30 mmHg, reflecting the actual collapse threshold of the arteriolar segment [15]. Quasi-no-flow estimates obtained using inspiratory-hold manoeuvres generally produce values in the 30–50 mmHg range, because flow is reduced but not abolished and intrathoracic pressure is elevated [14]. In contrast, arterial waveform modelling methods—whether based on aortic diastolic-decay fitting or on beat-to-beat MAP–flow regression—often give higher estimates (≈50–60 mmHg), because they infer a zero-flow intercept during ongoing flow rather than measure an actual collapse threshold [15,21]. These differences reflect methodology, not contradictory physiology. Table 1 outlines the various CCP estimation methods, their underlying assumptions, and the physiological constructs they represent.

A persistent source of confusion in the CCP literature is the assumption that all vascular collapse behaves like a Starling resistor. In reality, two fundamentally different mechanisms exist, ref. [18] and conflating them has led to many of the errors the present review aims to correct. Passive collapse occurs in highly compliant vessels such as veins or pulmonary vessels when external pressure exceeds intraluminal pressure. In this situation the vessel behaves as a collapsible tube: its radius decreases smoothly, flow varies approximately linearly with the difference between upstream pressure and external pressure, and downstream venous pressure becomes irrelevant only once external pressure exceeds downstream pressure, at which point a passive Starling resistor waterfall is formed. This is the classical Starling resistor behaviour that underpins zone-1 physiology in the lung and venous flow limitation under high PEEP or abdominal hypertension.

Active collapse, by contrast, occurs in arterioles where smooth-muscle tone generates wall tension that can exceed intraluminal pressure. Here the behaviour is not gradual but threshold-based. Experimental data from whole vascular beds and from microvascular models suggest that once local pressure falls below the critical closing pressure, flow does not enter a broad linear “waterfall” zone; it falls steeply toward very low values and remains minimal despite further reductions in pressure [16,17,18]. When upstream pressure rises again, flow increases sharply once the CCP threshold is exceeded. In other words, tone-dependent arteriolar collapse behaves predominantly as a valve-like process: conductance falls steeply as pressure approaches CCP, and once closure occurs, flow rapidly approaches zero. While narrow transitional regimes may exist near the threshold, available experimental data do not demonstrate a broad, sustained Starling resistor-type flow zone at physiologically relevant flows. 

This distinction is essential for interpreting haemodynamics in shock. The “waterfall zone” of the Starling resistor—where flow is governed by a continuous (P_upstream − P_external)/R relationship—exists only in passive collapse systems. It does not apply to arterioles exhibiting active, tone-dependent CCP behaviour. Treating arteriolar CCP as if it were a passive back-pressure leads directly to the common misconception that perfusion in collapsed beds follows a simple MAP − CCP relationship. In reality, pressure gradients are physiologically meaningful only in open vessels or passive collapse; in tone-dependent arterioles, CCP marks the boundary between open flow and cessation of flow, not a continuous inflow–outflow determinant.

Much of the historical confusion arose because classic analyses, particularly the influential work of Permutt and Riley [16], used the same language—“closing pressure” and “vascular waterfall”—to describe both passive external compression and active smooth-muscle-dependent closure. Their mathematical model applies strictly to passive collapsible-tube behaviour (P_ext–driven Starling resistors), but the terminology was extended to include tone-dependent arteriolar closure without a corresponding change in the underlying equations. Later authors, therefore, interpreted active arteriolar CCP as if it behaved like a Starling resistor with a continuous (P_upstream − Pcc) flow regime. This conflation is historically understandable but physiologically incorrect: tone-dependent arteriolar closure produces a threshold for flow cessation, not the sustained waterfall zone characteristic of passive collapse. The presence of a zero-flow intercept in these models remains a valid descriptor of flow cessation; however, it does not imply that arterioles exhibit a continuous collapsible-tube waterfall regime once tone-dependent closure occurs.

Recognising these fundamentally different mechanics prevents misinterpretation of both experimental CCP measurements and clinical haemodynamic data and clarifies why arteriolar collapse in shock behaves differently from venous or pulmonary collapsible-tube phenomena.

This misunderstanding leads to error in how CCP is used mathematically. Much of the literature treats CCP as if it were a continuous downstream pressure, inserting it into formulas such as “TPP = MAP − CCP” even when arterioles are open and autoregulated. This misrepresents a collapse threshold as if it were part of the normal pressure–flow relationship. CCP is not a standing back-pressure; it is a boundary condition that becomes relevant only once collapse has occurred [16]. In open vascular beds, flow remains governed by arterial–venous pressure differences, whereas once arterioles collapse, conductance falls to zero and no physiologically meaningful perfusion gradient exists. Using it outside that context attributes flow limitation to a variable that is physiologically irrelevant in open-flow conditions, becoming meaningful only after collapse.

These conceptual errors extend into the microcirculation. Some papers go further and apply CCP to the capillary bed itself—deriving expressions such as “capillary perfusion = CCP − Pms”—a category error that treats an arteriolar collapse threshold as if it governed capillary flow [12]. CCP is not the inflow pressure to the capillary compartment and cannot be paired with Pms in any physiologically meaningful way.

Importantly, CCP is usually dormant. Under healthy conditions, arteriolar pressure sits well above this threshold and the vessels remain open. CCP becomes physiologically relevant only when arterial pressure falls towards it, when CCP itself rises, or when both occur—circumstances common in advanced shock, over-zealous vasopressor therapy, or extrinsic compression.

## 5. The Vascular Waterfall—A Metaphor from Passive Collapse, Not a Property of Arterioles

The term vascular waterfall originated as a metaphor for passive collapsible-tube behaviour, as seen in veins under high external pressure or in zone-1 lung, and remains appropriate for passive external compression systems. In these settings, when external pressure exceeds downstream pressure, the vessel narrows, flow becomes independent of downstream pressure, and upstream pressure determines whether any flow occurs. This is a true passive Starling resistor waterfall because flow varies continuously with (P_upstream − P_external) [22].

Tone-dependent arteriolar collapse is fundamentally different. When pressure in an arteriole falls below its critical closing pressure (CCP), the smooth muscle generates enough wall tension to snap the vessel shut. Flow does not enter a continuous “waterfall zone”; it falls abruptly towards zero and remains minimal until pressure rises above the CCP threshold. This is more akin to a valve-like transition, not a collapsible-tube waterfall [18]. At physiologically relevant flows, there appears to be no important regime in which tone-dependent arteriolar flow behaves as a continuous (MAP − CCP) gradient. Available observations show steep, threshold-like transitions rather than a sustained waterfall-like flow zone. For this reason, the “vascular waterfall” metaphor should not be applied to arteriolar CCP physiology. The phenomenon of arteriolar collapse produces loss of conductance, not a Starling resistor flow regime.

So what is conditional about collapse? Although arterioles do not form a true waterfall, the state of collapse can still render downstream pressure irrelevant. Once an arteriole has closed, venous pressure no longer influences flow: reopening requires MAP to exceed CCP. This loss of downstream dependence is what has historically been likened to a waterfall, but the mechanism is fundamentally different from the passive collapsible-tube case. If this were a true waterfall, there would still be water falling from it; in tone-dependent arteriolar collapse, once CCP is reached, the flow largely stops, which is precisely why the waterfall metaphor is misleading. Thus, arteriolar collapse is a conditional state, a threshold event, not a structural waterfall and not the continuous pressure–flow behaviour of a Starling resistor.

Collapse is dynamic and heterogeneous. Whether a vascular bed is open or collapsed depends primarily on transmural pressure relative to its CCP, which is determined by local smooth-muscle tone and any external (perivascular) pressure acting on the vessel. Any factor that raises CCP (e.g., sympathetic tone, catecholamines, intracranial pressure, intrathoracic pressure, and interstitial oedema) or lowers local intraluminal pressure can push an arteriolar segment across its collapse threshold. Because these determinants vary across organs, different vascular beds reach their CCPs at different systemic pressures, producing patchy and heterogeneous closure during shock.

This matters for managing shock. Understanding collapse as a state, not a waterfall, clarifies why perfusion can fail despite apparently adequate MAP. When many arteriolar territories exceed their CCPs, they drop out of the conducting network entirely. In these segments, there is no meaningful “MAP − CCP” perfusion gradient—only a binary question of whether MAP exceeds the CCP threshold. Global variables such as MAP or SVR then describe only the surviving open beds, masking the underlying heterogeneity. When collapse is patchy, the macro-hemodynamic signal becomes uncoupled from the microcirculation, producing haemodynamic incoherence.

## 6. Where Collapse Actually Happens

Collapse does not occur everywhere in the circulation. It is almost exclusively a phenomenon of the arterioles, the small muscular vessels that sit just upstream of the capillary bed [18]. Arterioles actively generate tone, and thus tension, within their walls; when intraluminal pressure falls sufficiently, that tension can exceed the internal pressure and the vessel closes. This is where critical closing pressure arises [20]. 

In contrast, venous collapse in most physiological situations is passive: the segment narrows under external pressure, which increases resistance, but it does not normally impose a new fixed downstream pressure or redefine the driving gradient [18]. Flow continues to drain toward the right atrium, and the relevant back-pressure for systemic venous return remains RAP—not the external compression point.

A genuine venous Starling resistor—with a true “waterfall” regime in which flow is governed by (P_upstream − P_external) and becomes independent of downstream venous pressure—occurs only when external pressure exceeds downstream venous pressure (for example, with very high PEEP or marked abdominal hypertension). These are specialised circumstances and not the mechanism relevant to arterial CCP or to routine systemic venous return. Recent suggestions of a “venous waterfall” [12], therefore, misinterpret ordinary passive venous collapse as if it were equivalent to tone-dependent CCP in the arterioles.

The physiology makes the distinction unmistakable. If venous collapse in routine physiology behaved like a dominant opposing downstream pressure, then raising or lowering right atrial pressure would cease to influence venous return—yet this never happens: venous return reliably tracks the venular-to-RAP gradient. A true Starling resistor waterfall would also produce a plateau in the effective downstream pressure for flow, because once the vessel collapses, the external compression pressure, not RAP, would set the outflow pressure until RAP exceeded it. This plateau is never normally observed: CVP rises smoothly with congestion, and venous return continues to change with RAP rather than become independent of it, indicating that no passive waterfall has been created. Venous return does not become pressure-limited except under extreme external compression (e.g., very high PEEP or frank abdominal hypertension). In ordinary physiology, a narrowed or partially collapsed venous segment simply adds resistance; flow still drains toward the right atrium according to the upstream–downstream pressure difference.

These observations argue strongly against a dominant physiological “venous waterfall” in the systemic circulation. Routine venous return is governed by the Pms–RAP gradient through a mostly open, low-resistance network; only arterioles exhibit active, tone-dependent CCP behaviour, and even in that case, the Starling resistor waterfall model does not apply.

This distinction also explains why pressures lower than typical CCP values can exist within capillaries and venules without collapse: CCP refers specifically to the active tone-dependent closure threshold of arterioles, whereas other parts of the circulation may collapse at different pressures, but by entirely different—and passive—mechanisms.

Understanding where collapse occurs is key to interpreting vascular behaviour in shock. When arteriolar CCPs rise or local pressures fall, certain beds close and become isolated while others remain open. The result is heterogeneity of perfusion—the hallmark of haemodynamic incoherence [6].

## 7. Heterogeneity of Critical Closing Pressures Across Organs

Not all vascular beds behave alike when arterial pressure falls. Each organ’s CCP reflects the interaction between arteriolar tone, external tissue pressure, and local metabolic control [23]. This heterogeneity explains the characteristic sequence of organ dysfunction observed in shock [2].

Skin has the highest CCP. Its arterioles constrict strongly under sympathetic drive, diverting flow to vital organs. As MAP declines, cutaneous perfusion collapses early, producing the familiar pattern of cool, mottled extremities long before systemic hypotension becomes severe.

Splanchnic organs, including the gut and liver, have lower CCPs and remain patent until MAP falls much further. This relative preservation means that visceral flow can persist even when the skin and kidneys are already compromised.

The kidney occupies a more complex, dynamic position. At baseline its CCP is not especially high, but it is exceptionally sensitive to anything that raises the pressure needed to keep its arterioles open. Two mechanisms are particularly important:

### 7.1. Arteriolar Constriction

Afferent tone in the renal circulation is highly dynamic and central to the kidney’s early vulnerability in shock. Norepinephrine and sympathetic activation predominantly constrict the afferent arteriole, raising the local critical closing pressure (CCP) and the pressure required to maintain inflow [24]. At modest levels this may restore glomerular filtration by sustaining transglomerular pressure, but beyond a threshold, the rising CCP restricts total renal blood flow—producing the characteristic U-shaped renal response to catecholamines, where low doses recruit flow and high doses impair it despite higher systemic pressure.

Angiotensin II exerts mixed effects, constricting both afferent and efferent arterioles but more strongly on the efferent side. At physiological levels this maintains glomerular pressure without markedly raising afferent CCP, yet in high concentrations or when combined with catecholamines, it can further elevate CCP and limit inflow [25].

Vasopressin, in contrast, appears in many experimental models to act predominantly on the efferent arteriole [26]. By increasing downstream pressure within the glomerular capillaries, it may reduce the effective afferent CCP—functionally “splinting” the inflow segment in some settings and helping to restore perfusion when excessive catecholamine tone has closed it. However, human data are limited, and the net renal effect in septic shock is inconsistent; this mechanism should, therefore, be regarded as plausible but not proven.

### 7.2. External Constraint

The kidney’s encapsulation within Gerota’s fascia makes it an extremely low-compliance organ.

When venous pressure rises, even a small degree of interstitial oedema (nephrosarca) sharply increases interstitial hydrostatic pressure [27]. This pressure acts as an external compressive force on arterioles and capillaries, raising the effective CCP—a “capsular amplification” effect that, together with dynamic afferent tone, helps explain why the kidney becomes ischaemic early in shock despite apparently adequate systemic haemodynamics. In this specific context, the kidney approximates a true Starling resistor: a compliant vascular bed compressed within a rigid capsule, where rising interstitial pressure acts as an external constraint distinct from tone-dependent arteriolar CCP.

Direct measurements comparing organ-specific CCPs are scarce; most inferences arise from physiological reasoning, renal Doppler findings, and indirect haemodynamic studies.

Nevertheless, the pattern is consistent: the skin closes early, the kidney is dynamically fragile, and the gut and liver remain perfused longer. In contrast, cerebral and coronary circulations are the most robust, with autoregulatory mechanisms and structural protection that preserve flow until MAP falls extremely low—long after skin, renal, and splanchnic beds have begun to fail.

## 8. Systemic Vascular Resistance—Why the Numbers Mislead

Systemic vascular resistance is the most widely quoted measure of “afterload” in critical care, yet one of the most conceptually fragile. By definition, SVR = (MAP − RAP)/CO assumes the circulation behaves as a uniformly open network of parallel arteriolar resistors, all conducting flow. Under that assumption, the calculated SVR reasonably reflects aggregate arteriolar tone. This approximation holds only when every vascular bed remains open—that is, when Interface 2 (arteriolar→capillary continuity) is intact.

Once some beds collapse, the assumption breaks. A collapsed arteriolar segment does not adopt a different pressure–flow regime (there is no “MAP − CCP” perfusion in the open parts of the circulation). Instead, it simply drops out of the circuit and conducts no flow. Open beds continue to behave normally, with flow governed by MAP-to-RAP, while closed beds contribute zero conductance. The global circulation, therefore, no longer behaves like a single equivalent resistor; it behaves like a parallel network in which multiple branches have been removed. In physical terms, systemic vascular resistance is the inverse of total vascular conductance. In a parallel network, total conductance equals the sum of individual bed conductances (G_total = G_1_ + G_2_ + …). When a vascular bed collapses, its conductance approaches zero and it is effectively removed from the circuit. Total conductance, therefore, falls, and calculated SVR rises, even if arteriolar tone in the remaining open beds is unchanged. Therefore, the calculated SVR increasingly reflects a mathematical artefact of the surviving open pathways, not a direct index of systemic vasomotor tone.

**Figure 1:**
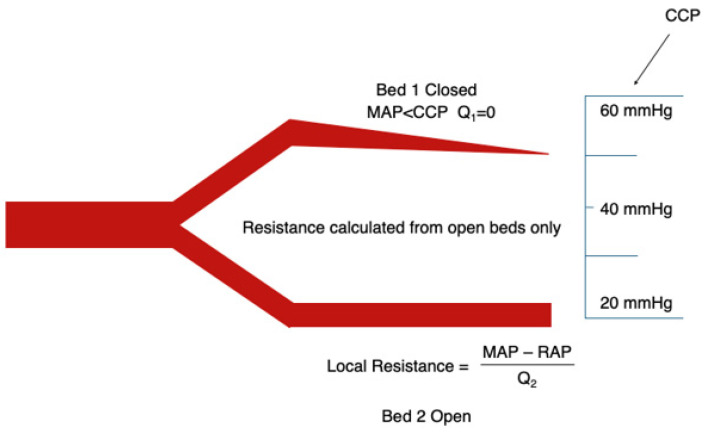
Heterogeneous critical closing pressures in parallel vascular beds.

**Figure 2:**
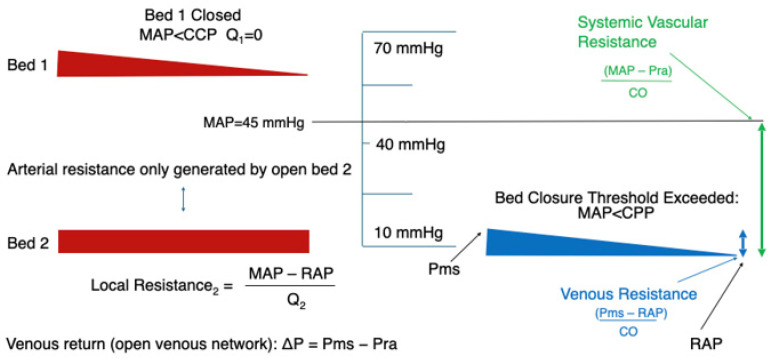
Critical closing pressure and its effect on SVR.

**Figure 3:**
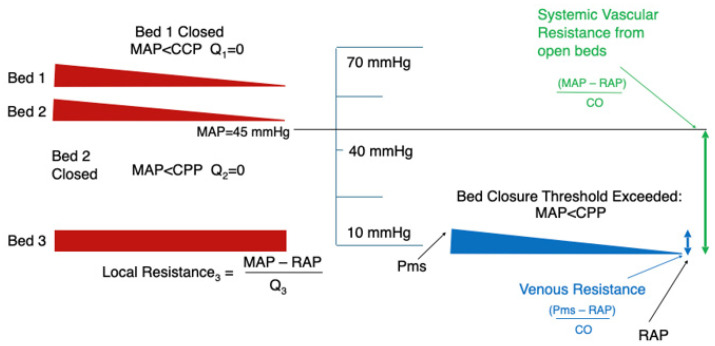
Sequential recruitment of vascular beds as arterial pressure rises.

**Figure 4:**
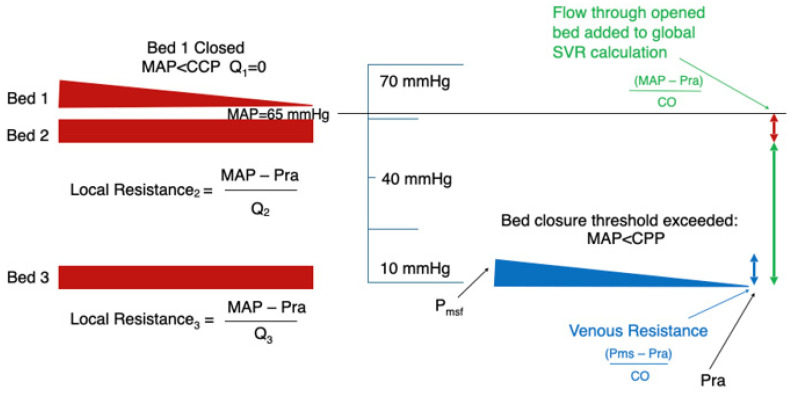
Further recruitment of vascular beds.

Figure 1, Figure 2, Figure 3 and Figure 4 illustrate this behaviour: when one vascular territory is open and another is collapsed, SVR reflects only the conductance of the open pathways. Depending on how many beds remain patent, and how many have closed behind their CCP thresholds, SVR may appear high, normal, or even low. None of these necessarily correspond to actual vasoconstriction. This explains how SVR may appear normal despite profound regional hypoperfusion or paradoxically high even when vasomotor tone is not excessive.

Two vascular beds arranged in parallel with different critical closing pressures (CCP). Bed 1 has a lower CCP and remains open at a given MAP; Bed 2 has a higher CCP and collapses when MAP falls below its CCP. When a bed is open, flow is determined by the MAP–RAP gradient and arteriolar resistance. When a bed is collapsed, flow falls to zero rather than following a “MAP − CCP” gradient. CCP, therefore, determines whether a bed conducts flow, not the pressure gradient through it.

SVR reflects only the conductance of vascular beds that remain open. When a bed collapses (MAP < CCP), its conductance drops to zero and it no longer contributes to total flow. The measured SVR, therefore, becomes the inverse of the summed conductance of the surviving open beds. SVR may appear normal or elevated despite widespread microvascular closure—not because of increased vasomotor tone, but because fewer pathways remain in the parallel network.

At low mean arterial pressure (MAP ≈ 45 mmHg), only the lowest-CCP vascular territory remains open; the intermediate- and high-CCP beds have collapsed and are isolated behind their own collapse thresholds. Flow is restricted to the single patent bed even though MAP remains above venous pressure.

When MAP increases to ≈65 mmHg, the intermediate-CCP bed reopens while the high-CCP bed remains closed.

These panels illustrate that vascular beds reopen only once MAP exceeds their individual CCP thresholds, making perfusion recovery a sequential, not all-or-none, process.

Maas and colleagues demonstrated collapse in situ using inspiratory-hold manoeuvres to estimate CCP [14]. Above the closing pressure, the pressure–flow relationship was approximately linear, permitting extrapolation to a zero-flow intercept; flow then fell steeply as pressure approached CCP, consistent with a threshold-dominated loss of conductance rather than a sustained waterfall regime. This abrupt transition is entirely consistent with Burton’s earlier mechanical analysis, which showed that muscular vessels become unstable once transmural pressure falls below a critical value [18]. This strongly suggests that CCP becomes relevant only once arterioles collapse—not during normal autoregulated flow and not during the pressure-dependent (but still open) phase that precedes collapse. Despite this, some later interpretations treated the so-called “waterfall” as if it were a persistent structural feature and even subdivided SVR into “arterial” and “venous” resistances, as if all segments behaved linearly regardless of collapse. Such approaches apply only to no-flow or highly constrained states; they do not describe the mixed open–closed physiology of shock.

Clinically, these mixed states are almost certainly the norm in shock. Some organs remain open and perfused; others sit behind closed arterioles. In such scenarios the numerical SVR loses its physiological meaning. A high SVR does not imply excessive vasoconstriction—it may reflect loss of conducting pathways. A normal SVR does not imply adequate perfusion—it may simply reflect preserved flow in the few beds that remain open. A low SVR is not proof of global vasodilation—it may occur when enough beds are open even though others remain collapsed.

The therapeutic implication is straightforward. Interventions aimed at “optimising SVR”—raising MAP, escalating vasopressors, or chasing a target afterload—are often ineffective because they do not address the true problem: failure of Interface 2, where macro- and microcirculation lose continuity. The goal is not to normalise SVR, but to reopen the closed segments by lowering excessive tone, relieving external compression, or correcting volume overload. Only then does the circulation return to a regime in which SVR again reflects something physiologically coherent. In this sense, SVR-driven management fails not because the physiology is complex, but because it is irreducibly individual: the relevant question is which beds are open, which are closed, and why.

## 9. When CCP Becomes Flow-Limiting

Critical closing pressure (CCP) is not continuously flow-determining. Forces tending to collapse vessels are always present, but CCP becomes relevant only when those forces are strong enough to disrupt the normal pressure–flow relationship and parts of the vascular network collapse. This can occur in three principal ways, through excess tone, external constraint, or profound hypotension, which frequently coexist in critically ill patients.

### 9.1. Tone-Driven Elevation of CCP (“Pressor Problem”)

Excess sympathetic or pharmacologic vasoconstriction stiffens arteriolar smooth muscle, raising the pressure needed to keep small vessels open. Once local transmural pressure falls below this threshold, arterioles close and flow becomes threshold-limited by CCP rather than downstream pressure [17]. Typical contexts include any form of shock and receiving high-dose vasopressors, as well as acute surges in endogenous sympathetic tone. This represents Interface 2 failure—the macro-to-micro disconnection caused by tone-driven collapse.

### 9.2. Compression-Driven Elevation of CCP (“Constraint Problem”, Interface 3 and 4)

Here, the closing force arises externally. Raised venous pressure—often from right heart dysfunction, elevated intrathoracic pressure, or pulmonary vascular load at Interface 4—leads to interstitial oedema, especially in encapsulated organs such as the kidney or brain. The resulting increase in tissue hydrostatic pressure elevates the effective CCP and impedes inflow despite normal MAP. Even a small volume increase can sharply raise interstitial pressure, making this a quintessential Interface 3 failure, though its trigger may originate one step upstream.

### 9.3. Profound Hypotension (“Low-Drive Problem”, Interface 1)

Collapse can also occur when arterial pressure falls below the local CCP, as in extreme hypotension [14,15]. Here, flow stops not because CCP has risen, but because MAP has fallen beneath the pressure required to keep vessels open. This occurs well below the lower limit of autoregulation, when even fully dilated arterioles can no longer sustain flow. It represents failure at Interface 1—ventricular–arterial uncoupling producing inadequate upstream pressure, seen in profound shock or cardiac arrest, where vessels remain closed until arterial pressure is restored above the CCP threshold. As illustrated in Figure 2, as mean arterial pressure rises above successive critical closing pressures, vascular beds reopen one by one—explaining why perfusion recovery during resuscitation occurs in a graded, rather than uniform, fashion.

### 9.4. Mixed States Are the Rule

Most ICU patients lie somewhere between these extremes: regional over-tone in some beds, oedematous compression in others, and global hypotension adding to both. The result is patchy perfusion, haemodynamic incoherence, and misleading global variables with clinical meaning determined by the individual distribution of open and closed beds.

## 10. Recognising CCP-Limited Flow at the Bedside

Collapse of parts of the microcirculation is rarely seen directly, but it leaves clear footprints.

When macro-haemodynamics appear stable yet the tissues look poorly perfused, suspect that one or more interfaces have failed. Haemodynamic incoherence, the coexistence of reassuring global values (such as MAP or cardiac output) with clinical hypoperfusion, is the hallmark. It signals that perfusion pressure has fallen below, or closing pressure has risen above, the threshold for flow in some vascular beds.

1. Bedside clues:
2. Cold, mottled peripheries despite “adequate” MAP ≥ 65 mmHg;
3. Capillary refill > 3 s;
4. Oliguria despite normal or high CO and MAP;
5. Perfusion worsening as vasopressor dose or positive fluid balance rises;
6. High central venous saturation with high lactate, implying shunted or stagnant microcirculatory flow.

- Clinical patterns of collapse:
- The three principal mechanisms of CCP elevation have distinct bedside patterns:
- Tone-driven (Interface 2)—cold, mottled extremities and prolonged CRT despite adequate MAP.
- Compression-driven (Interface 3)—oedematous tissues or organs (e.g., kidney) with rising venous pressures.
- Low-drive (Interface 1)—global hypotension with uniformly weak perfusion.

In reality, these states often coexist, producing mixed or fluctuating patterns of haemodynamic incoherence.

Where available, microcirculatory assessment tools may provide supportive evidence of impaired flow continuity. Techniques such as sublingual sidestream dark-field imaging, near-infrared spectroscopy, peripheral perfusion indices, or renal Doppler resistive indices can help corroborate clinical suspicion of regional hypoperfusion. These modalities are best viewed as adjuncts rather than prerequisites: the core diagnosis of haemodynamic incoherence remains clinical and physiological, based on discordance between global haemodynamic variables and tissue perfusion.

## 11. Therapeutic Implications—Restoring Flow Continuity

Treatment aims not simply to raise pressure but to re-couple the interfaces and eliminate un-perfused capillary beds. CCP can rise from tone, volume, or external compression; therapy must lower the right component rather than chase the wrong number. Examples are given in Table 2.

Recent applied physiology studies have attempted to “restore” the vascular waterfall as a therapeutic target. Andrei et al. [3] observed improved perfusion after norepinephrine in vasoplegic patients, while Liu et al. [4] reported similar findings with β-blockade in septic shock. In both cases, the apparent benefit was attributed to an increased gradient between critical closing pressure and mean systemic pressure (Pms)—interpreted as a “restored” waterfall. Yet in both cohorts, mean arterial pressure remained well above CCP, confirming that flow never approached collapse and that a true vascular waterfall state is unlikely to have existed. Because CCP and Pms were derived from zero-flow extrapolations, their rise simply marked a theoretical closing threshold, not a real opposing pressure. In the norepinephrine study, improvement likely resulted from MAP moving further above that threshold and, in the β-blocker study, from better ventricular–arterial coupling and oxygen balance. These examples illustrate how therapies may improve perfusion by moving the circulation away from closed capillary beds, not by recreating them. The practical aim is to restore open-flow continuity, not to “manipulate” a waterfall.

A similar issue arises in recent proposals to manipulate a putative “venous waterfall,” where it has been suggested that increasing CVP might not impair venous return because flow would remain independent of RAP until a venous “critical pressure” is reached [12]. This conclusion follows only if passive venous narrowing is treated as a dominant downstream pressure, which it is not in normal systemic veins; in practice, raising CVP consistently worsens organ congestion, reduces the arteriolar–venular pressure drop, and increases interstitial pressure, thereby elevating effective CCP and impairing perfusion rather than improving it.

Importantly, CCP-guided reasoning does not imply lowering MAP targets indiscriminately or reducing vasopressors in all forms of shock. Arterial pressure must remain above organ-specific autoregulatory limits, particularly for the brain and heart, and time-critical priorities such as haemorrhage control, coronary perfusion, and severe vasoplegia take precedence. Within these safety boundaries, excessive vasopressor-driven tone, venous congestion, or external compression may raise arteriolar CCP and worsen microvascular dropout. In such settings, escalating MAP alone may fail to restore perfusion. CCP-based reasoning, therefore, complements, rather than replaces, established MAP safety thresholds by helping identify when restoring flow continuity requires reducing excessive tone or constraint rather than further pressure escalation. This approach aligns with contemporary randomised evidence showing that personalised haemodynamic resuscitation targeting capillary refill time normalisation improves patient-centred outcomes in early septic shock, reinforcing that restoration of tissue-level perfusion, not further escalation of global pressure targets, should guide therapy [28].

## 12. Case Examples (Hypothetical)

### 12.1. Case 1|The Over-Pressured Mixed Distributive and Cardiogenic Shock

A middle-aged man with septic shock is resuscitated to a MAP of 70 mmHg on escalating norepinephrine. Cardiac output is assumed to be adequate—no echocardiography has yet been performed—but in reality, he has developed unrecognised septic cardiomyopathy. SVR is high, CVP has climbed during resuscitation, and despite “normal” numbers, the patient remains cool and mottled with a capillary refill time of six seconds and declining urine output.

The apparent haemodynamic stability is deceptive: the seemingly adequate MAP is sustained largely by high afterload and venous pressure, not by forward flow. Excessive vasoconstriction has raised local CCP above the pressure required to keep small vessels open, while elevated CVP compounds the problem indirectly by increasing venous and interstitial pressures. This external constraint further elevates effective CCP and narrows the perfusion gradient. Many microvascular beds are now functionally excluded behind collapse arterioles despite an apparently satisfactory blood pressure.

A focused echocardiogram later confirms a low-output state. Norepinephrine is reduced, a small dose of inotrope is introduced, and MAP is now maintained with higher flow and lower CVP and SVR—reflecting an exchange of pressure for flow. Capillary refill and urine output improve. Here, success comes not from adding pressure but from restoring flow continuity—lowering CCP, widening the gradient, and re-establishing perfusion through previously closed vascular beds.

### 12.2. Case 2|The Oedematous Kidney

A patient recovering from septic shock is now stable on a norepinephrine infusion titrated to maintain the conventional target MAP of 65 mmHg. Cardiac output is high and the calculated systemic vascular resistance (SVR) is low—an expected finding after aggressive fluid resuscitation. The patient is markedly peripherally oedematous, yet without cardiac failure or markedly elevated right atrial pressure (RAP ≈ 10 mmHg, near the flat portion of the Starling curve). Despite apparently excellent macro-haemodynamics, urine output is falling and creatinine is rising.

In this setting, the problem is not tone but pressure from within the organ itself.

Excess interstitial fluid has accumulated in the renal parenchyma, which is confined by a non-compliant capsule. Even a modest increase in interstitial volume sharply raises interstitial hydrostatic pressure, effectively increasing renal CCP. Although MAP meets standard targets, the true perfusion gradient across the renal microcirculation is critically reduced. Diuretic therapy and a period of negative fluid balance gradually relieve the interstitial pressure, and urine output improves without any change in vasopressor support.

Here, as in the previous case, the limiting factor was CCP, not MAP, but in this instance, it rose through oedema rather than arteriolar tone.

## 13. Conclusions and Future Directions

The behaviour of the circulation near its limits is rarely linear. As arterial pressure, vascular tone, and tissue pressure interact, parts of the vascular tree can drop out of circuit entirely once the critical closing pressure (CCP) is reached. Recognising this phenomenon reframes how we interpret the familiar numbers of critical care.

Perfusion is not governed by a single pressure or resistance but by the continuity of flow across the interfaces of the circulation—ventricle to arteries, arteries to capillaries, and capillaries to veins. When these interfaces fail—through loss of driving pressure, excessive arteriolar tone, or external compression—local pressures fall to, or are forced above, their critical closing thresholds. Once arterioles close, the macro- and microcirculations become uncoupled at Interface 2, disconnecting portions of the circulation and producing the haemodynamic incoherence so often seen at the bedside: reassuring monitors beside a hypoperfused patient.

Clinically, the message is simple: when the numbers and the tissues disagree, trust the tissues. Restoring flow requires lowering CCP when it is excessive, raising inflow pressure only when it is truly low, and reducing external or venous constraint when the circulation is compressed. Therapy succeeds not when MAP or SVR reach a target, but when perfusion reconnects from pump to tissue with interventions guided by the individual pattern of vascular recruitment and constraint rather than by uniform haemodynamic thresholds.

Although the framework presented here draws on consistent physiological principles, it is built from indirect evidence, whole-bed flow studies, microvascular models, and inferences from pressure–flow behaviour, rather than direct single-arteriole recordings in humans. Existing data strongly support a steep, threshold-like transition at arteriolar collapse, and they do not demonstrate a sustained Starling resistor-type waterfall at physiologically relevant flows. But the exact shape of the transition zone, and its heterogeneity across organs and disease states, remain incompletely defined. These uncertainties do not weaken the conceptual distinction between passive collapsible-tube behaviour and tone-dependent active collapse; rather, they point to where empirical refinement is still needed.

Much of the historical confusion surrounding CCP arose because different pressure–flow frameworks—autoregulation, venous return, microvascular flow, and Starling resistor theory—were often applied independently. When their assumptions were combined without reconciliation, threshold behaviour was mistaken for a continuous downstream pressure, and active arteriolar closure was conflated with passive collapsible-tube mechanics. By revisiting the governing physics at each vascular segment and integrating them through the four-interface model, we clarify why these contradictions emerged and how they can be resolved. What appeared as inconsistent physiology is better understood as mismatched analytical lenses; when aligned, the role of CCP becomes coherent, bounded, and clinically meaningful.

Future research should, therefore, focus on identifying CCP-limited states at the bedside, validating microcirculatory or ultrasound surrogates for collapse, and exploring therapies that restore coupling between interfaces rather than merely normalising macro-variables. Understanding when and where CCP becomes relevant may prove as transformative for perfusion management as the recognition of autoregulation was for cerebral physiology.

## Figures and Tables

**Table 1:** Methods used to estimate critical closing pressure (CCP) or related zero-flow intercepts. Different techniques rely on distinct physiological assumptions and, therefore, estimate different constructs; values are not interchangeable and should be interpreted in the context of the method used.

Method	How CCP Is Estimated	Key Assumptions	What It Represents	Typical Reported Range	Major Pitfalls/Limitations	Notes
True no-flow measurements (circulatory arrest/vascular occlusion)	Direct observation of arterial pressure at which flow ceases	Complete cessation of flow; minimal wave effects	Actual mechanical collapse threshold of the arteriolar segment	~20–30 mmHg	Rarely feasible in humans; reflects extreme conditions	Gold standard for true CCP
Inspiratory-hold/low-flow extrapolation	Linear regression of pressure–flow relationship during reduced-flow states	Linear P–Q relationship while vessels remain open; quasi-steady conditions	Estimated collapse threshold under reduced but non-zero flow	~30–50 mmHg	Influenced by intrathoracic pressure; extrapolated zero-flow intercept	Valid approximation when assumptions hold
Arterial waveform modelling (diastolic decay, Pzf)	Mathematical fitting of arterial pressure decay	Lumped-parameter models; constant compliance	Effective zero-flow intercept, not direct mechanical collapse	~45–60 mmHg	Model-dependent; not equivalent to arteriolar CCP	Often misinterpreted as true CCP
Beat-to-beat MAP–flow regression	Extrapolation of MAP–CO relationship to zero flow	Linear MAP–flow relationship across operating range	Functional zero-flow pressure during ongoing circulation	Variable	Conflates resistance, compliance, and tone	Descriptive, not mechanistic
Derived CCP–Pms gradients (“waterfall” formulations)	Subtraction of estimated CCP from MAP or Pmsf	Assumes CCP behaves as a continuous downstream pressure	Theoretical construct, not a physical pressure	Not applicable	Physiologically invalid in open vascular beds	Source of major conceptual errors

**Table 2:** Interface-based strategy for restoring flow continuity.

Interface	Typical Presentation	Mechanism	Therapeutic Focus
1. Pump—pipes	Falling LVEF or low stroke volume with high afterload.	Ventricular–arterial uncoupling, excessive tone.	Deescalate pressor; consider inotrope; moderate afterload.
2. Macro—micro	Cool, mottled, CRT > 3 s, despite MAP target.	Arteriolar collapse→high CCP.	Reduce tone (less NE or add vasodilator); accept slightly lower MAP if flow improves.
3. Capillary—venous	Oliguria + oedema ± RAP = 10–12 mmHg	Raised interstitial pressure→external CCP.	Diuresis/UF; reduce venous pressure or excessive PEEP; avoid further fluids.

## Data Availability

No new data were created or analysed in this study.

## Abbreviations

The following abbreviations are used in this manuscript:

CCP	Critical Closing Pressure
MAP	Mean Arterial Pressure
TPP	Tissue Perfusion Pressure
Pms	Mean Systemic Pressure
SVR	Systemic Vascular Resistance
RAP	Right Atrial Pressure
CO	Cardiac Output
PEEP	Positive End-Expiratory Pressure
RV	Right Ventricle/Right Ventricular
LV	Left Ventricle/Left Ventricular
CRT	Capillary Refill Time
ICU	Intensive Care Unit
